# Beyond Traditional Methods: Deep-Learning Machines Empower Fingerroot (*Boesenbergia rotunda*)-Extract Production with Superior Antioxidant Activity

**DOI:** 10.3390/foods13172676

**Published:** 2024-08-25

**Authors:** Padej Pao-la-or, Kakanang Posridee, Pussarat Buranakon, Jittra Singthong, Jirawan Oonmetta-Aree, Ratchadaporn Oonsivilai, Anant Oonsivilai

**Affiliations:** 1 School of Electrical Engineering, Institute of Engineering, Suranaree University of Technology, Nakhon Ratchasima 30000, Thailand; padej@sut.ac.th; 2 School of Food Technology, Institute of Agricultural Technology, Suranaree University of Technology, Nakhon Ratchasima 30000, Thailand; posridee.ka@gmail.com (K.P.); p.buranakon@gmail.com (P.B.); 3 Department of Agro-Industry, Faculty of Agriculture, Ubon Ratchathani University, Warinchamrap, Ubon Ratchathani 34190, Thailand; jittrawara@gmail.com; 4 Food Science and Technology Program, Faculty of Science and Technology, Nakhon Ratchasima Rajabhat University, Nakhon Ratchasima 30000, Thailand; ojirawan@gmail.com; 5 Health and Wellness Research Unit, Suranaree University of Technology, Nakhon Ratchasima 30000, Thailand

**Keywords:** deep-learning machine, response-surface methodology, fingerroot, optimization, percentage yield, antioxidant activity

## Abstract

This study investigated the impact of drying parameters on the quality of fingerroot (*Boesenbergia rotunda*) extract, focusing on phenolic compounds, flavonoids, and antioxidant activity. A Box–Behngen design was employed to evaluate the effects of maltodextrin concentration, inlet temperature, and outlet temperature on the extract’s properties. The highest total phenolic content (18.96 µg of GAE/mg extract) and total flavonoid content (33.52 µg of GE/mg extract) were achieved using 20% maltodextrin, a 160 °C inlet temperature, and an 80 °C outlet temperature. Antioxidant activity, measured by DPPH and FRAP assays, was also influenced by drying parameters. Stepwise regression analysis revealed that maltodextrin concentration significantly affected all responses, while the inlet temperature had no significant effect. The outlet temperature significantly influenced FRAP activity. The developed mathematical models accurately predicted experimental values, validating the effectiveness of the RSM and Deep-Learning Machine. Optimal drying conditions for maximizing phenolic compounds were determined to be 20% maltodextrin, a 150 °C inlet temperature, and a 70 °C outlet temperature, resulting in TPC 15.33 µg of GAE/mg extract, TF 28.75 µg of GE/mg extract, IC_50_ value of 3.99 µg/mL, FRAP value at 4.44 µmoL Fe^2+^/mg extract of phenolic content, and 18.96 µg of the GAE/mg extract. Similar conditions were found to be optimal for maximizing flavonoid content. These findings provide valuable insights for optimizing the drying process of fingerroot extract to preserve its bioactive compounds and enhance its potential applications.

## 1. Introduction

*Boesenbergia rotunda*, a medicinal plant from the ginger family, has been extensively studied for its bioactive compounds and therapeutic properties. Researchers have employed various solvents, such as methanol, ethanol, water, and their combinations, along with extraction techniques like maceration, Soxhlet extraction, ultrasound-assisted extraction, and supercritical fluid extraction, to obtain these compounds [1,2,3,4,5,6]. The major bioactive compounds extracted include flavonoids (e.g., pinostrobin, pinocembrin, and alpinetin), chalcones (e.g., Panduratin A, Panduratin B, and Panduratin C), and essential oils (e.g., camphene, β-pinene, and borneol) [5,7,8]. These compounds have exhibited various bioactivities, such as antioxidant, anti-inflammatory, antimicrobial, and anticancer properties [9,10,11]. Optimization techniques, like response-surface methodology (RSM), have been employed to enhance the extraction yield and bioactivity [2,5], while purification methods, such as column chromatography, preparative HPLC, and crystallization, have been used to isolate and purify specific bioactive compounds from the crude extracts [7,8].

The extracts obtained from *Boesenbergia rotunda*, particularly those containing flavonoids, chalcones, and essential oils, have exhibited significant antioxidant properties in various studies. Cheng et al. [9] isolated and characterized antioxidant compounds from *B. rotunda* extracts, finding that the extracts contained substantial amounts of phenolic compounds and flavonoids, which contributed to their strong antioxidant activity. Morikawa et al. [11] investigated the structure–activity relationships of pimarane-type diterpenes from *B. rotunda*, and some of these diterpenes displayed notable antioxidant effects, suggesting their potential as natural antioxidant agents. Although studying a different plant species (*Alpinia zerumbet*), Elzaawely et al. [3] employed similar extraction methods and solvents as those used for *B. rotunda*, demonstrating the ability of these techniques to obtain antioxidant-rich extracts from related plants in the ginger family. These findings collectively indicate the significant antioxidant potential of the bioactive compounds extracted from *B. rotunda*, warranting further exploration of these extracts as natural sources of antioxidants for various applications.

Deep-learning and machine-learning techniques have been increasingly applied in various areas of biological sciences, including genomics and bioinformatics for genome sequencing and assembly, gene-expression analysis, protein structure prediction, and biomarker discovery [12,13]; proteomics and metabolomics for protein–protein interaction prediction, metabolic pathway analysis, and small molecule identification and characterization [14,15]; molecular modeling and drug discovery for the virtual screening of drug candidates, protein–ligand interactions and binding affinity predictions, and quantitative structure–activity relationship (QSAR) modeling [16,17]; biomedical imaging for image segmentation and analysis, disease diagnosis and classification, and microscopy image analysis [18,19]; and systems biology and computational biology for modeling biological networks and pathways, simulation of biological systems, and integration and analysis of multi-omics data [20,21]. Deep-learning architectures like convolutional neural networks (CNNs), recurrent neural networks (RNNs), and deep-belief networks have been successfully applied in these areas, often outperforming traditional machine-learning methods in tasks such as pattern recognition, image analysis, and sequence analysis [22,23]. While the provided references do not cover these applications, deep learning and machine learning are rapidly emerging fields in biological sciences, offering powerful tools for data analysis, modeling, and prediction in various domains.

Deep-learning models are built on artificial neural networks with multiple interconnected layers, enabling them to learn hierarchical representations of data and automatically extract increasingly complex features from raw inputs, making them effective for tasks like image recognition, natural language processing, and speech recognition [22,23]. The backpropagation algorithm, a supervised learning technique that calculates error gradients to adjust network parameters, is a fundamental component that allows for the efficient training of these multi-layer networks [24]. Nonlinear activation functions, such as the rectified linear unit (ReLU), introduce nonlinearity into the models, enhancing their ability to learn complex, nonlinear relationships within the data [25]. Additionally, deep learning leverages the principle of transfer learning, where pre-trained models can be fine-tuned on specific tasks, transferring knowledge gained from large datasets to related domains with limited data, a process known as feature transferability [26]. Furthermore, deep-learning models can scale to handle large datasets and benefit from parallel computing architectures like GPUs, accelerating the training process and enabling the development of increasingly complex and powerful models [23].

Artificial intelligence (AI) and machine learning (ML) have emerged as indispensable tools for optimizing food-drying processes. ML models, ranging from shallow- to deep-learning techniques, have effectively addressed challenges like uneven drying, nutrient degradation, and high energy consumption. By intelligently configuring these models and integrating them with real-time measurement systems, industries can achieve dynamic optimization and automated decision-making. Moreover, AI’s ability to predict drying times and analyze energy usage patterns offers significant potential for resource efficiency. Despite these advancements, challenges such as data quality, integration complexity, and ethical considerations persist [27].

Deep learning (DL) offers significant advantages in food-drying optimization. DL models can accurately predict key parameters like drying time, energy consumption, and product quality, enabling efficient process control. By integrating DL with real-time measurements, drying parameters can be dynamically adjusted to maintain optimal conditions. Furthermore, DL-based algorithms can automate decision-making and reduce human errors, improving process efficiency. Additionally, DL can identify opportunities to optimize resource usage, such as minimizing energy consumption and waste, contributing to sustainable food production [28,29,30,31].

Deep learning, a subset of machine learning, has emerged as a promising tool for optimizing food-drying processes. These studies demonstrate the potential of DL to enhance various aspects of food drying, including modeling, prediction, and process control. By leveraging the power of AI, DL can address challenges such as energy efficiency, product quality, and operational optimization, leading to more sustainable and efficient food production [28,29,30,31].

This research aims to enhance the antioxidant properties of *Boesenbergia rotunda* extract through an optimized drying process. By employing Response Surface Methodology (RSM) and Dynamic Linear Models (DLM), we sought to identify the optimal drying conditions that maximize antioxidant activity while minimizing product degradation. This study contributes to the development of efficient and effective drying techniques for preserving bioactive compounds in natural products.

## 2. Materials and Methods

All chemicals of analytical grade, including 1,1-diphenyl-2-picryhydrazyl free radical (DPPH), 2,4,6-tri(2-pyridyl)-s-triazine (TPTZ), ferric, chloride-6-hydrate, ferrous, sulphate 7-hydrate, acetate buffer pH 4.6, gallic acid, Folin–Ciocalteu phenol reagent, anhydrous sodium carbonate (Na_2_CO_3_), catechin, sodium nitrite (NaNO_2_), and aluminum chloride (AlCl_3_), were obtained from Sigma–Aldrich Co. (St. Louis, MO, USA). Solvents such as ethanol were purchased from Mallinckrodt–Baker (Phillipsburg, NJ, USA).

### 2.1. Fingerroot Powder Preparation

Fingerroot powder was prepared from *Boesenbergia rotunda* specimens collected in Nakhon Ratchasima Province, Thailand, during November 2023. The collected finger roots were thoroughly rinsed to remove any adhering dirt or debris and subsequently subjected to a drying process at 60 °C for 24 h to remove moisture content. After drying, the fingerroot was ground into a fine powder using an Ultra Centrifugal Mill Model ZM-1000 (Retsch, Hann, Germany), and the resulting powder was sieved through a mesh size of 0.2 mm to obtain a uniform particle-size distribution. Finally, the sieved finger root powder was stored in vacuum-sealed packages at a temperature of −20 °C until further use, ensuring the maintenance of its quality and preventing potential degradation during storage. This standardized procedure facilitated the preparation of a homogeneous fingerroot powder from *Boesenbergia rotunda*, suitable for various applications, such as extraction of bioactive compounds or incorporation into formulations.

### 2.2. Fingerroot Extraction

The ethanol-extraction method for fingerroot (*Boesenbergia rotunda*) followed a similar procedure to the water extraction, except 57% ethanol was used as the solvent instead of water. One gram of finger root powder was accurately weighed and extracted three times with 10 mL of 57% ethanol. The combined extract was then adjusted to a final volume of 50 mL. Aliquots of 2 mL were taken from the adjusted extract and transferred into individual test tubes. Unlike water extraction, the ethanol extracts were dried using a vacuum dryer instead of freeze-drying. The dried extracts obtained from the vacuum drying process were stored at −20° C until further use or analysis [24].

### 2.3. Total Phenolic Contents

The total soluble phenolic constituents of the extracts were determined using the Folin–Ciocalteu method, with gallic acid employed as the standard reference compound [3,26]. In this procedure, 20 μL of the crude extract solution, standard gallic acid solution, or blank solvent were added to a 1.5 mL cuvette, followed by the addition of 1.58 mL of deionized water and 100 μL of the Folin–Ciocalteu reagent. The mixture was thoroughly mixed and incubated for 5 min at room temperature. After incubation, 300 μL of a 2% (*w*/*v*) sodium carbonate (Na_2_CO_3_) solution were introduced, and the mixture was allowed to stand at room temperature for 2 h to facilitate the development of the blue-colored complex resulting from the reduction of the Folin–Ciocalteu reagent by the phenolic compounds present in the sample. Subsequently, the absorbance of the solution was measured at a wavelength of 765 nm using a spectrophotometer. The total soluble phenolic content was quantified by establishing a calibration curve with known concentrations of the gallic acid standard, and the results were expressed as gallic acid equivalents (GAE) based on the linear regression equation obtained from the calibration curve [3,4,26].

### 2.4. Total Flavonoid

The total flavonoid contents of the extracts were determined using the aluminum chloride colorimetric method [4,31]. In this assay, 250 μL of the crude extract solution, standard solution, or blank were added to a 1.5 mL cuvette, followed by the addition of 1.25 mL of deionized (DI) water and 75 μL of 5% sodium nitrite (NaNO_2_) solution. The mixture was incubated for 6 min at room temperature. After incubation, 150 μL of 10% aluminum chloride (AlCl_3_) solution were introduced, followed by the sequential addition of 0.5 mL of 1 M sodium hydroxide (NaOH) and 275 μL of DI water. The resulting mixture was then incubated for an additional 5 min to allow the development of the pink-colored flavonoid–aluminum complex. Subsequently, the absorbance of the solution was measured at a wavelength of 510 nm using a spectrophotometer. The total flavonoid contents were quantified by establishing a calibration curve with known concentrations of the catechin standard, and the results were expressed as catechin equivalents based on the linear regression equation obtained from the calibration curve.

### 2.5. DPPH Assay

The DPPH (2,2-diphenyl-1-picrylhydrazyl) free-radical scavenging activity of Thai basil extracts (water, ethanol, and ethyl acetate), as well as the positive controls butylated hydroxytoluene (BHT) and ascorbic acid, were evaluated using a DU 800 Spectrophotometer (Beckman Coulter, Brea, CA, USA) according to the method described by Oonsivilai et al. [5]. An aliquot (100 μL) of the extract at varying concentrations or the positive control solutions prepared in methanol was mixed with 1.9 mL of a methanolic DPPH solution. The reaction mixtures were incubated in the dark for a specified duration, during which the DPPH radical was reduced by the antioxidant compounds present in the extracts or positive controls, resulting in a discoloration of the DPPH solution. The decrease in absorbance at a specific wavelength (typically 515–520 nm) was measured spectrophotometrically and used to calculate the percentage of DPPH radical scavenging activity. The IC_50_ values, representing the concentration of the extract or positive control required to scavenge 50% of the DPPH radicals, were determined by nonlinear regression analysis using SigmaPlot 9.1 software (Systat Software Inc., Chicago, IL, USA) [5].

### 2.6. FRAP Assay

The ferric-reducing antioxidant power (FRAP) assay was performed according to the method described by Oonsivilai et al. [5]. The FRAP reagent was prepared by combining an acetate buffer (pH 3.6), a 10 mM solution of 2,4,6-tripyridyl-s-triazine (TPTZ) in 40 mM hydrochloric acid, and a 20 mM ferric chloride solution in the ratio of 10:1:1 (*v*/*v*/*v*), respectively. An aliquot (50 μL) of the extract was added to 1.5 mL of the freshly prepared FRAP reagent, and the mixture was incubated for 4 min at room temperature to facilitate the reduction of the ferric–tripyridyltriazine complex to the ferrous form by the antioxidant compounds present in the extract. After the incubation period, the absorbance of the reaction mixture was measured at 593 nm using a spectrophotometer. A calibration curve was constructed using ferric sulfate solutions of known concentrations ranging from 100 to 2000 μM. The FRAP values of the extracts were calculated from the linear regression equation obtained from the calibration curve and expressed as millimoles of ferric equivalents per gram of dry plant material [5,26].

### 2.7. Spray Drying Condition

#### 2.7.1. Experimental Design

Response Surface Methodology (RSM), a well-established optimization technique, was employed to identify the optimal drying conditions that maximize the extraction yield, total phenolic contents, total flavonoid contents, and antioxidant activities [31,32,33]. Three independent variables—maltodextrin concentration (X_1_: 20, 25, and 30% *w*/*v*), inlet temperature (X_2_: 140, 150, and 160 °C), and outlet temperature (X_3_: 70, 80, and 90 °C)—were investigated for their influence on the dependent variable, extraction yield. A Box–Behnken design was utilized to randomize the experimental order, and the resulting data were analyzed using regression analysis [34,35]. The independent variables are in Table 1.

#### 2.7.2. Experimental Validation of the Optimal Conditions

A model-driven optimization approach was employed to maximize the extraction yield, total phenolic contents (TPC), total flavonoid contents (TFC), and antioxidant activities as measured by DPPH and FRAP assays. The validity of the model was subsequently assessed by performing three independent extraction experiments under the predicted optimal conditions, which deviated from those employed for model development.

#### 2.7.3. Deep-Learning Machine Modeling

Deep-learning models are built on artificial neural networks with multiple interconnected layers, enabling them to learn hierarchical representations of data and automatically extract increasingly complex features from raw inputs, making them effective for tasks like image recognition, natural language processing, and speech recognition [22,23]. The backpropagation algorithm, a supervised learning technique that calculates error gradients to adjust network parameters, is a fundamental component allowing for efficient training of these multi-layer networks [35]. Nonlinear activation functions, such as the rectified linear unit (ReLU), introduce nonlinearity into the models, enhancing their ability to learn complex, nonlinear relationships within the data [36]. Additionally, deep learning leverages the principle of transfer learning, where pre-trained models can be fine-tuned on specific tasks, transferring knowledge gained from large datasets to related domains with limited data, a process known as feature transferability [37]. Furthermore, deep-learning models can scale to handle large datasets and benefit from parallel computing architectures like GPUs, accelerating the training process and enabling the development of increasingly complex and powerful models [23].

### 2.8. Statistical Analysis

All experiments were performed in triplicate, and mean values (on a dry basis) with standard deviations were reported. The experimental data were analyzed using an analysis of variance (ANOVA). SPSS^®^ software Version 17 (SPSS Inc., Chicago, IL, USA) was used to perform all statistical calculations.

## 3. Results

### 3.1. Validation of the Experimental Design

This study investigated the influence of drying parameters on the quality of a fingerroot extract initially obtained using 57% ethanol. A factorial design was employed, analyzing the effects of maltodextrin concentration (20, 25, 30% *w*/*v*), inlet temperature (140, 150, 160 °C), and outlet temperature (70, 80, 90 °C) on the extract’s content of phenolic compounds, flavonoids, and its free-radical scavenging activity.

The total phenolic contents in the dried fingerroot extract ranged from 5.63 to 18.96 μg of GAE/mg extract. The combination of 20% maltodextrin, 160 °C inlet temperature, and 80 °C outlet temperature yielded the highest phenolic content. Similarly, the total flavonoid contents varied between 5.55 and 33.52 μg of GE/mg extract, with the same drying conditions (20% maltodextrin, 160 °C inlet temperature, 80 °C outlet temperature) producing the most significant amount.

Free-radical scavenging activity, measured by the DPPH method, ranged from 0.67 to 4.22 μg/mL of extract. Interestingly, drying conditions with either 20% or 30% maltodextrin, inlet temperatures of 140 or 150 °C, and outlet temperatures of 70 or 90 °C resulted in the highest IC_50_ and FRAP values (1.28 to 4.829 µmol Fe^2^⁺/mg extract). However, the drying condition with 20% maltodextrin, 160 °C inlet temperature, and 80 °C outlet temperature exhibited the strongest FRAP value. For detailed results of all experimental conditions, please refer to Table 2 and Table 3.

When taking the total amount of phenolic compounds obtained from dried fingerroot extracts, the relationship is found with the independent variables using stepwise regression analysis, which can display results in the form of regression analysis. Details are shown in Table 4, Table 5 and Table 6.

Table 2 and Table 3 present the fingerroot experimental conditions with the predicted experimental values, and deep-learning machine results of TPC, TF, DPPH, and FRAP assays, as well as the extraction yield of the recovered bioactive compounds from fingerroot.

The dried extract yield ranges from 23.34 ± 0.68% (run 15, 30%, 140 °C, 70 °C) to 67.29 ± 0.46% (run 5, 30%, 150 °C, 70 °C). The TPC ranged from 5.63 µg of the GAE/mg extract (run 13, 25%, 160 °C, 90 °C) to 18.96 µg of the GAE/mg extract (run 9, 20%, 160 °C, 80 °C). The TF ranged from 5.55 µg of the GE/mg extract (run 13, 25%, 160 °C, 90 °C) to 33.52 µg of the GE/mg extract (run 9, 20%, 160 °C, 80 °C). The antioxidant activity evaluated by the DPPH assay ranged between IC50 4.22 µg/mL (run 1, 20%, 140 °C, 80 °C) and IC50 0.67 µg/mL (run 10 and 5, 30%, 150 °C, 70 °C). In what concerns the FRAP assay, results ranged between 1.28 µmol of the FSE/mg extract (run 6, 25%, 150 °C, 80 °C) and 4.83 µmol of the FSE/mg extract (run 9, 20%, 160 °C, 80 °C). Interestingly, the results obtained for the drying condition at maltodextrin 20%, inlet temperature 150 °C, and outlet temperature 70 °C, were similar for both antioxidant activity responses. In addition, the inlet temperature and outlet temperature did not affect the DPPH and FRAP responses. The highest DPPH response (1.24 µg/mL) was achieved at maltodextrin 20%, inlet temperature 150 °C, and outlet temperature 70 °C.

The experimental values for the four responses were similar (*p* > 0.05) to the respective predicted ones and DLM data, allowing the validation of the experimental design and demonstrating the effectiveness and adequacy of the RSM and Deep-Learning Machine (Table 4, Table 5 and Table 6).

### 3.2. Analysis of Response Surface

The independent variable X_1_ (maltodextrin concentration, % *w*/*v*) showed a significant effect (*p* < 0.05) on TF, DPPH, FRAP, and yield response, whereas the independent variable X_2_ (Inlet temperature, °C) showed no significant effect (*p* > 0.05) on all responses. In addition, the X_3_ (Outlet temperature, °C) showed a significant effect (*p* < 0.05) on FRAP response. Furthermore, the quadratic term for X_1_ and X_2_ showed a significant effect on Y4 response (FRAP). The R^2^ is useful for checking the model fitness, being in this study classified as strong for FRAP response (R^2^ adjust was relatively close to 1) (Table 4, Table 5 and Table 6). Considering all response-surface models, the “lack of fit” was significant (*p* < 0.05) for yield response but not significant (*p* > 0.05) for TPC, TF, DPPH, and FRAP response.

#### 3.2.1. Fitting Model

The parameters of the equation were determined through linear and multiple regression analysis of the experimental results.

Total phenolic contents = 45.27942 − 0.406750X_1_ − 0.085X_2_ − 0.129375X_3_

Total flavonoid contents = 1335.61375 − 5.83042X_1_ − 18.213 X_2_ + 3.64454 X_3_ − 0.0532 X_1_X_2_ + 0.01615 X_1_X_3_ + 0.02125 X_2_X_3_ + 0.227583 X_1_^2^ + 0.059071 XX_2_^2^ − 0.046654 X_3_^2^

DPPH = 15.31492 − 0.18525X_1_ − 0.0305X_2_ − 0.043875X_3_

FRAP = 284.89475 − 0.412750 X_1_ − 3.19655 X_2_ − 0.803425 X_3_ − 0.006935 X_1_X_2_ − 0.003285 X_1_X_3_ + 0.002467 X_2_X_3_ + 0.031535 X_1_^2^ + 0.010484 X_2_^2^ − 0.002874 X_3_^2^

Yield = −95.52675 + 2.46725X_1_ + 0.386250X_2_ + 0.341625X_3_

**Figure 1:**
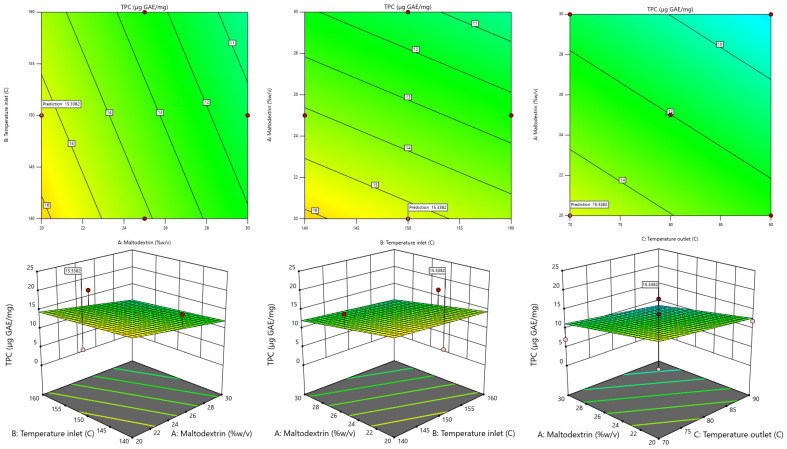
Response-surface plots depicting the interactive effects of total phenolic contents.

**Figure 2:**
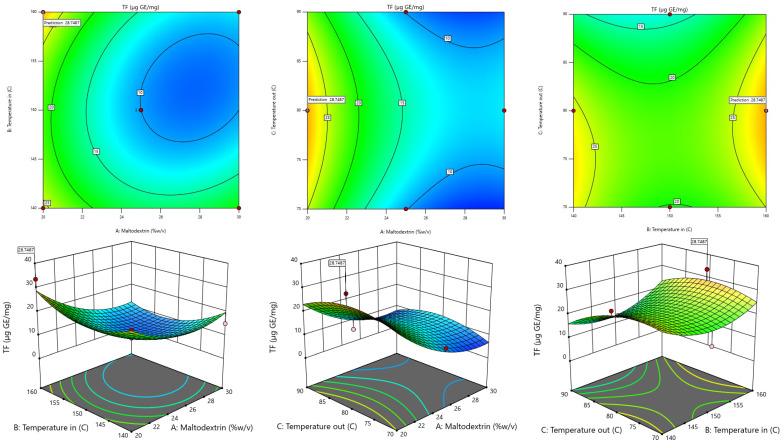
Response-surface plots depicting the interactive effects of total flavonoid contents.

**Figure 3:**
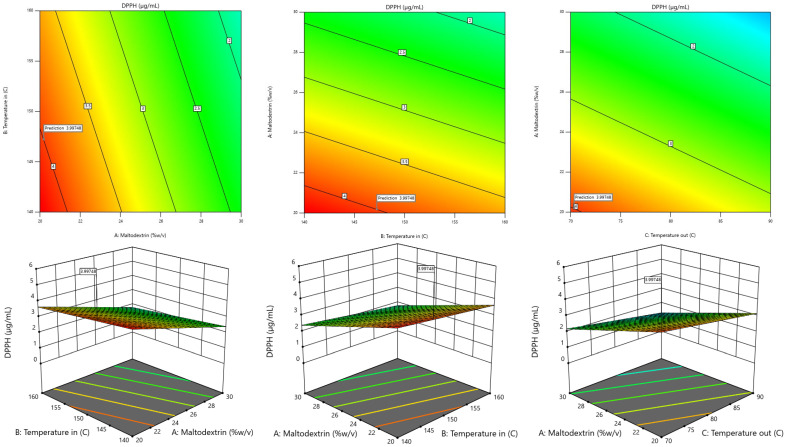
Response-surface plots depicting the interactive effects of IC_50_ value.

**Figure 4:**
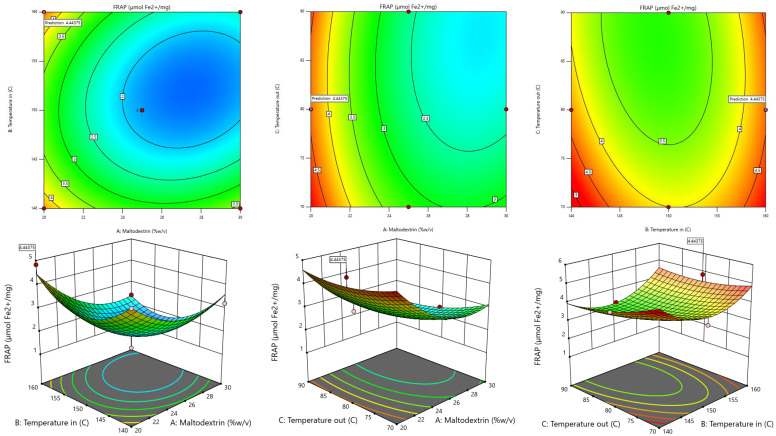
Response-surface plots depicting the interactive effects of FRAP value.

**Figure 5:**
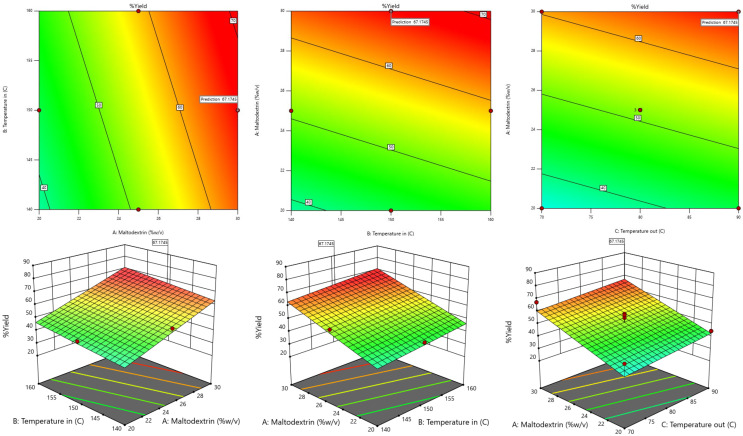
Response-surface plots depicting the interactive effects of yield percentage.

The RSM model was used to generate 3D contour plots in order to represent graphically the relationship between independent (maltodextrin concentration, inlet temperature, outlet temperature) and dependent variables (TPC, TF, DPPH, FRAP, Yield). As shown in Figure 1, Figure 2, Figure 3, Figure 4 and Figure 5.

#### 3.2.2. Total Phenolic Contents

When taking the TPC value obtained from dried fingerroot extract, the relationship with the independent variables is found by using Stepwise regression analysis, which can display results in the form of regression analysis. Details are shown in Table 4.

The table showing the results of the regression analysis determines the second-order mathematical model. This can be used to create a mathematical model or mathematical equation for predicting the dependent variable (response) by taking the values of the factors obtained from the analysis of the coefficients of the regression equation of the said dependent variable and writing them in the form The linear model equation is shown in Equation (1), which has an R^2^ value of 0.2502.

Y1 = 45.27942 − 0.406750X_1_ − 0.085X_2_ − 0.129375X_3_ (1)

The reduced second-order mathematical model in Equation (1) is used as a function to determine the appropriate conditions for drying using a linear model solution, which helps to find the optimum condition that provides the highest amount of total phenolic contents. The results of the experiment show that the drying conditions were at a maltodextrin concentration of 20% *w*/*v*, an inlet temperature of 160 °C, and an outlet temperature of 80 °C, which results in the highest amount of phenolic compound When compared with the values calculated from model predictions at the same condition, it was found that the number of phenolic compounds was equal to 18.96 µg GAE/mg of dried fingerroot extract. When calculating the most suitable conditions for drying to obtain the highest number of phenolic compounds, a prediction by the model found that a maltodextrin concentration of 20% *w*/*v*, an inlet temperature of 150 °C, and an outlet temperature of 70 °C resulted in the number of phenolic compounds equal to 15.34 µg GAE/mg of dried fingerroot extract.

#### 3.2.3. Total Flavonoid Contents

When taking the TF value obtained from dried fingerroot extract, the relationship with the independent variables was found using stepwise regression analysis, which can display results in the form of regression analysis. Details are shown in Table 4.

The results of the regression analysis can determine the second-order mathematical model, which can be used to create a mathematical model or mathematical equation for predicting the dependent variable (response) by taking the values of the factors obtained from the analysis of the coefficients of the regression equation of the said dependent variable and writing them in the form. The quadratic model equation is shown in Equation (2), which has an R^2^ value of 0.7975.

Y1 = 1335.61375 − 5.83042X_1_ − 18.213 X_2_ + 3.64454 X_3_ − 0.0532 X_1_X_2_ + 0.01615 X_1_X_3_ + 0.02125 X_2_X_3_ + 0.227583 X_1_^2^ + 0.059071 X_2_^2^ − 0.046654 X_3_^2^ (2)

The reduced second-order mathematical model in Equation (2) is used as a function to determine the appropriate conditions for drying. A non-linear model solution can be used to find the optimum condition that provides the highest amount of total flavonoid contents. The results of the experiment show that the drying conditions were at a maltodextrin concentration of 20% *w*/*v*, an inlet temperature of 160 °C, and an outlet temperature of 80 °C, resulting in the highest number of flavonoid compounds. When compared with the values calculated from model predictions under the same conditions, the number of flavonoid compounds was found to be 33.52 µg GE/mg of dried fingerroot extract. When calculating the optimum drying conditions that resulted in the highest flavonoid content from a prediction by the model, it was found that the drying conditions at a maltodextrin concentration of 20% *w*/*v*, an inlet temperature of 160 °C, and an outlet temperature of 80 °C in the number of flavonoid compounds equaled to 28.75 µg GE/mg of dried fingerroot extract.

#### 3.2.4. DPPH Assay

When taking the IC_50_ value obtained from dried fingerroot extract, the relationship with the independent variables was found using stepwise regression analysis, which can display results in the form of regression analysis. Details are shown in Table 5.

Based on the table showing the results of the regression analysis, the second-order mathematical model can be determined and used to create a mathematical model or mathematical equation for predicting the dependent variable (response) by taking the values of the factors obtained from the analysis of the coefficients of the regression equation of the said dependent variable and writing them in the form. The linear model equation is shown in Equation (3), which has an R^2^ value of 0.4202.

Y1 = 15.31492 − 0.18525X_1_ − 0.0305X_2_ − 0.043875X_3_ (3)

The reduced second-order mathematical model in Equation (3) is used as a function to determine the appropriate conditions for drying using a linear model solution and find the optimum condition with the highest IC_50_ value. The results of the experiment show that the drying conditions using maltodextrin concentrations of 20 % *w*/*v*, inlet temperatures of 140, and an outlet temperature of 80 °C result in the highest IC_50_ value. When compared with the values calculated from model predictions at the same conditions, it was found that the IC_50_ value was equal to 4.22 µg/mg of dried fingerroot extract, which, when calculating the most suitable conditions for drying, resulted in the highest IC_50_ value from the model prediction. It was found that the drying condition at a maltodextrin concentration of 20.191% *w*/*v*, an inlet temperature of 147.227 °C, and an outlet temperature of 70.352 °C resulted in an IC_50_ value of 3.99748 µg/mg of dried fingerroot extract.

#### 3.2.5. FRAP Assay

When taking the FRAP value obtained from dried fingerroot extract, the relationship with the independent variables was found using stepwise regression analysis, which can display results in the form of regression analysis. Details are shown in Table 5.

Based on the table showing the results of the regression analysis, the second-order mathematical model can be determined and used to create a mathematical model or mathematical equation for predicting the dependent variable (response) by taking the values of the factors obtained from the analysis of the coefficients of the regression equation of the said dependent variable and writing them in the form The quadratic model equation is shown in Equation (4), which has an R^2^ value of 0.9063.

Y1 = 284.89475 − 0.412750 X_1_ − 3.19655 X_2_ − 0.803425 X_3_ − 0.006935 X_1_X_2_ − 0.003285 X_1_X_3_ + 0.002467 X_2_X_3_ + 0.031535 X_1_^2^ + 0.010484 X_2_^2^ − 0.002874 X_3_^2^ (4)

The reduced second-order mathematical model in Equation (4) is used as a function to determine the appropriate conditions for drying. A non-linear model solution is used to find the optimum condition with the highest FRAP value. The results of the experiment showed that the drying conditions were at a maltodextrin concentration of 20% *w*/*v*, an inlet temperature of 160 °C, and an outlet temperature of 80 °C, resulting in the highest FRAP value. When compared with the values calculated from model predictions at the same conditions, it was found that the FRAP value was equal to 4.829 µmol Fe^2+^/mg dried fingerroot extract when calculating the most appropriate conditions for drying, resulting in the highest FRAP value from the model prediction. It was found that drying conditions with a maltodextrin concentration of 20% *w*/*v*, an inlet temperature of 160 °C, and an outlet temperature of 80 °C resulted in a FRAP value of 4.44 µmol Fe^2+^/mg of substance dried fingerroot extract.

#### 3.2.6. Yield Percentage and Moisture Contents

The moisture content of the dried fingerroot and fingerroot extract was determined to be 6.49 ± 0.13% and 5.73 ± 0.14%, respectively. When taking the yield percentage obtained from dried fingerroot extract, the relationship with the independent variables can be found using stepwise regression analysis, which can display results in the form of regression analysis. Details are shown in Table 6.

From the table showing the results of the regression analysis, the second-order mathematical model can be used and determined to create a mathematical model or mathematical equation for predicting the dependent variable (response) by taking the values of the factors obtained from the analysis of the coefficients of the regression equation of the said dependent variable and writing them in the form The linear model equation is shown in Equation (5), which has an R^2^ value of 0.9063.

Y1 = −95.52675 + 2.46725X_1_ + 0.386250X_2_ + 0.341625X_3_ (5)

The reduced second-order mathematical model in Equation (5) is used as a function to determine the appropriate conditions for drying using a linear model solution, and the optimum condition with the highest percentage yield is found. The results of the experiment showed that the drying conditions were at a maltodextrin concentration of 30% *w*/*v*, an inlet temperature of 160 °C, and an outlet temperature of 80 °C in the highest percentage yield. When compared with the values calculated from model predictions at the same conditions, it was found that the percentage yield was equal to 65.89 when calculating the most appropriate conditions for drying, resulting in the highest percentage yield from the model prediction. It was found that drying conditions with a maltodextrin concentration of 30% *w*/*v*, an inlet temperature of 150 °C, and an outlet temperature of 90 °C resulted in a percentage yield of 67.18.

### 3.3. Data Acquisition and Preprocessing for Deep-Learning Machines

**Figure 6:**
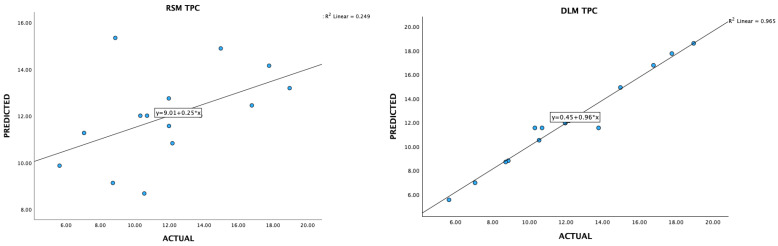
A plot of predicted and experimental values for total phenolic contents from *Boesenbergia rotunda* crude extract using RSM and DLM.

**Figure 7:**
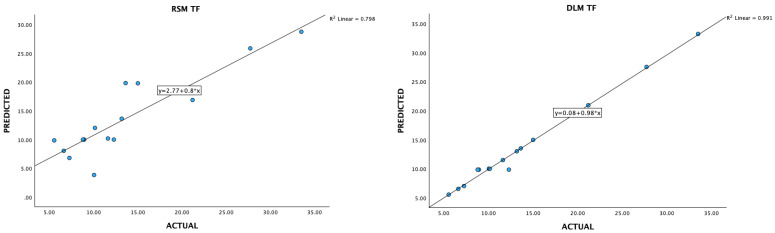
A plot of predicted and experimental values for total flavanoid contents from *Boesenbergia rotunda* crude extract using RSM and DLM.

**Figure 8:**
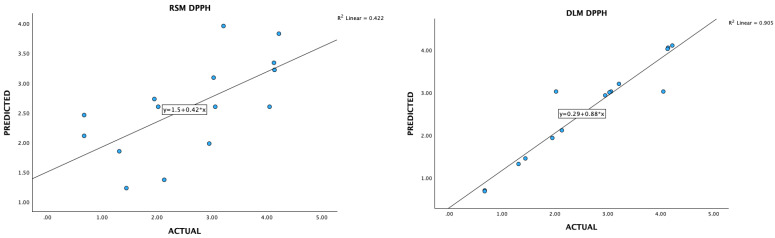
A plot of predicted and experimental values for DPPH radical scavenging activity from *Boesenbergia rotunda* crude extract using RSM and DLM.

**Figure 9:**
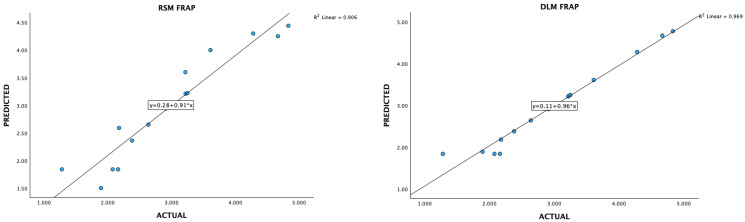
A plot of predicted and experimental values for FRAP activity from *Boesenbergia rotunda* crude extract using RSM and DLM.

**Figure 10:**
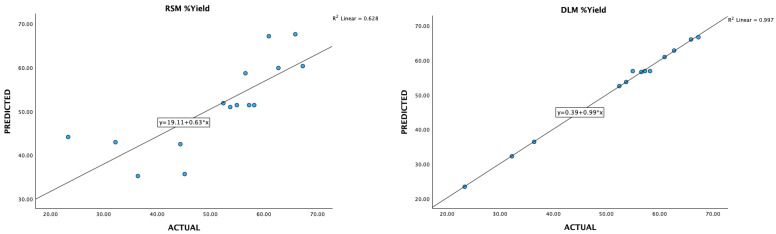
A plot of predicted and experimental values for yield percentage from *Boesenbergia rotunda* crude extract using RSM and DLM.

Optimizing extraction conditions within a laboratory setting can be a cumbersome and time-consuming endeavor, particularly when multiple variables are involved, each possessing various levels. The Box–Behnken design serves as a prevalent strategy to streamline this process by minimizing the required number of experiments. However, recent advancements in deep-learning machines (DLMs) have demonstrated their superiority over Response Surface Methodology (RSM) in achieving superior optimization outcomes (Figure 6, Figure 7, Figure 8, Figure 9 and Figure 10).

## 4. Discussion

Building upon research on fingerroot (Alpinia galanga), this study investigates two crucial stages: extraction and drying. The first study focused on optimizing the microwave-assisted extraction (MAE) process to maximize the total phenolic and flavonoid contents in fingerroot extracts. Here, water extraction yielded significantly higher phenolic content (up to 18.96 μg of GAE/mg extract) compared to ethanol extraction, aligning with findings by [38,39]. Additionally, optimized MAE conditions (20% (*w*/*v*) maltodextrin, microwave 500 W, 160 °C inlet temperature, 80 °C outlet temperature) resulted in the highest overall phenolic contents.

From previous research, this study explores the impact of drying parameters on a pre-extracted fingerroot extract obtained using 57% ethanol. Interestingly, a combination of 20% maltodextrin, a 160 °C inlet temperature, and an 80 °C outlet temperature during the drying process resulted in the highest levels of preserved phenolic and flavonoid compounds. However, other drying conditions (variations in maltodextrin concentration and inlet and outlet temperatures) yielded extracts with the strongest free-radical scavenging activity (FRAP values). This observation underscores the intricate relationship between drying parameters and the retention of bioactive compounds within the fingerroot extract. These findings align with the ongoing efforts to identify factors influencing the potential health benefits of fingerroot, as previously explored by Kanjanasirirat et al. [38].

This research investigates a critical aspect of fingerroot preservation—the drying process—with the aim of maximizing its health benefits. While this research lies on optimizing drying parameters (such as maltodextrin concentration and inlet/outlet temperature) to retain the highest levels of beneficial compounds (phenolics, flavonoids) and free-radical scavenging activity within pre-extracted fingerroot extracts, a complementary study by Fahrudin et al. [40] explores the impact of different drying methods (freeze drying, oven drying, sun drying) on the overall quality of fingerroot powder. Notably, [40] investigate aspects such as moisture content, color, solubility, and morphology, which provide a broader perspective on the influence of drying on fingerroot beyond the specific bioactive compounds targeted in the present study. This comprehensive approach underscores the importance of considering various drying techniques and their multifaceted effects on fingerroot quality.

This research investigates the optimal drying conditions for preserving the valuable bioactive compounds in fingerroot extract (likely fingerroot) using a spray-drying technique. The study identified that a combination of 20% maltodextrin, 160 °C inlet temperature, and 80 °C outlet temperature yielded the highest levels of phenolics, flavonoids, and FRAP value, indicating strong overall antioxidant activity. Interestingly, a broader range of drying conditions (20–30% maltodextrin, 140–150 °C inlet temperature, 70–80 °C outlet temperature) resulted in the highest IC50 value, suggesting maximized free-radical scavenging activity. Furthermore, the use of response-surface methodology allowed for slight adjustments in drying parameters to target specific bioactive compounds.

While the reference itself describes the current study, the DPPH assay method used to measure IC50 values likely originates from the work of [41]. Other references like [41,42,43,44], although not directly cited here, offer valuable insights into spray-drying optimization for various materials. By comparing their findings on factors like carrier material (maltodextrin) and temperature with this study on fingerroot extract, future research can establish broader trends in spray drying-optimization for bioactive compound preservation.

Overall, this study effectively utilizes spray drying to optimize fingerroot extract production by identifying drying conditions that maximize the retention of valuable bioactive compounds and antioxidant activity. This approach aligns with existing research and paves the way for further exploration of spray-drying parameters for different materials.

Researchers are exploring Deep Learning (DL) for drying process optimization. In Zhang et al. [45], a Deep-Learning Machine (DLM) was applied to identify drying conditions that maximize the yield and bioactivity of extracted fingerroot compounds. In this study, researchers focused on developing a Deep-Learning Model Predictive Control (DL-MPC) system to optimize drying efficiency in a complex paddy drying system. While this fingerroot experiment aimed to improve product quality, the paddy drying research prioritized reducing computational cost and achieving faster control compared to traditional methods. Both studies leverage deep learning for drying process optimization but in different ways. The fingerroot experiment employed a Deep-Learning Machine (DLM) to predict optimal drying conditions based on factors like temperature and maltodextrin concentration. The good agreement between predicted and actual results validates the DLM’s effectiveness for this specific application. Meanwhile, the paddy drying research proposes a Deep-Learning Model Predictive Control (DL-MPC) system. This system uses deep-learning models to forecast future drying behavior and optimize control decisions, like adjusting air temperature, in real time. Notably, the research emphasizes the significant reduction in computational time compared to traditional methods, making DL-MPC suitable for online control in industrial drying settings. Both studies achieved positive outcomes using deep learning for drying process optimization. The fingerroot experiment identified ideal drying conditions (20% maltodextrin, 150 °C inlet temperature, 70 °C outlet temperature) that maximized extract yield and antioxidant activity. This success highlights the potential of Deep-Learning Machines (DLMs) for optimizing fingerroot extraction processes. In the paddy drying research, researchers implemented a Deep-Learning Model Predictive Control (DL-MPC) system for a complex multistage drying system. This system achieved significant improvements in computational speed compared to traditional methods. Field tests further confirmed the effectiveness of the DL-MPC controller in maintaining consistent moisture content in the paddy and achieving smoother control over the drying process compared to manual adjustments. Despite both studies utilizing deep learning for drying process optimization, their focus and goals differed. The fingerroot experiment aimed to maximize the yield and bioactivity (TPC, TF, DPPH, FRAP) of extracted compounds from a specific botanical material—fingerroot. Here, the DLM identified optimal drying conditions based on factors like temperature and maltodextrin concentration. In contrast, the paddy drying research dealt with optimizing efficiency in a complex multistage drying system for a staple crop—paddy rice. Their primary goal was to reduce computational cost and improve control speed compared to traditional methods. Here, the Deep-Learning Model Predictive Control (DL-MPC) system focused on optimizing drying efficiency (reducing drying time and energy consumption) while ensuring the paddy rice maintains the desired moisture content.

Traditional drying methods, such as oven drying, spray drying, and freeze drying, often struggle to provide precise control over drying conditions, leading to the potential degradation of bioactive compounds and suboptimal product quality. Empirical methods, relying on trial-and-error experiments, can be time-consuming and may not offer a systematic understanding of the complex relationships between process variables, hindering the optimization of drying processes.

Advanced optimization techniques offer promising solutions for complex drying processes. Artificial Neural Networks (ANNs) can model nonlinear relationships but require substantial data and are often difficult to interpret. Genetic Algorithms (GAs) explore a wide range of solutions but can be computationally intensive and may get stuck in local optima. Response-Surface Methodology (RSM) effectively models nonlinear processes but is limited to a smaller number of variables.

RSM-DLM offers several advantages over traditional methods for drying process optimization. By combining the strengths of RSM and DLM, RSM-DLM provides a more comprehensive approach to modeling and optimizing dynamic processes. Additionally, DLM’s ability to incorporate uncertainty and variability makes the model more robust to real-world conditions. The flexibility of RSM-DLM allows it to handle a variety of process variables and constraints. Comparative studies, if available, can further demonstrate the effectiveness of RSM-DLM compared to other methods in optimizing drying processes.

## 5. Conclusions

By studying the appropriate drying conditions of dried fingerroot extract, it was found that factors affecting physical and chemical properties, with a maltodextrin concentration level of 30% (*w*/*v*), will result in the highest amount of dried fingerroot extract. A maltodextrin concentration level of 20% (*w*/*v*) will make the dried fingerroot extract have the most yellow color, and an outlet temperature of 90 °C will result in the moisture content, while the amount of free water makes the value minimal. In terms of pH, alkalinity and solubility were not significantly different (*p* > 0.05).

The study of the most suitable conditions for drying fingerroot extract found that drying with a spray dryer obtained the highest amount of active ingredients. Also, the response-surface methodology was used to find the most suitable conditions for drying the fingerroot extract. The results from the research can be summarized as follows: The drying conditions that resulted in the highest number of phenolic compounds were a maltodextrin concentration of 20% (*w*/*v*), an inlet temperature of 160 °C, and an outlet temperature of 80 °C, which corresponded to the number of flavonoid compounds and the FRAP value.

Meanwhile, the drying conditions using maltodextrin concentrations of 20 and 30% (*w*/*v*), inlet temperatures of 140 and 150 °C, and outlet temperatures of 70 and 80 °C resulted in the IC_50_ value. Also, a prediction by mathematical model found that drying conditions using a maltodextrin concentration of 20% (*w*/*v*), an inlet temperature of 160 °C, and an outlet temperature of 70 °C resulted in the highest number of phenolic compounds.

Meanwhile, the drying condition using a maltodextrin concentration of 20% (*w*/*v*), an inlet temperature of 160 °C, and an outlet temperature of 78.9 °C resulted in the highest flavonoid compound content. The drying conditions are most suitable for the antioxidant capacity. Based on the prediction using the model, the details are as follows. The drying condition using a maltodextrin concentration of 30% *w*/*v*, an inlet temperature of 160 °C, and an outlet temperature of 70 °C resulted in the highest IC_50_ value, while the drying condition at the concentration level of maltodextrin 20% *w*/*v*, an inlet temperature of 140 °C, and an outlet temperature of 70 °C resulted in the highest FRAP value.

When comparing the stability results of important substances in dried fingerroot extracts by drying with a spray dryer, it was found that the number of important substances in the dried fingerroot extract decreased, while the yield of the dried fingerroot extract increased. Compared to drying with a vacuum oven and freeze dryer, the physical characteristics of dried fingerroot extracts dried with a vacuum oven and freeze dryer are lumpy, hard, and insoluble in water but soluble in ethanol. The physical characteristics of the white fingerroot extract that are dried with a spray dryer will be in the form of a powder that is soluble in water. Therefore, it is suitable for application in functional food products.

## Figures and Tables

**Table 1:** Encoded and coded levels of independent variables used in the experimental design.

Symbols	Independent Variables	Coded Levels		
		−1	0	1
*X*_1_	Maltrodextrin (% *w*/*v*))	20	25	30
*X*_2_	Inlet Temperature (°C)	140	150	160
*X*_3_	Outlet Temperature (°C)	70	50	90

**Table 2:** Experimental and predicted values of TPC and TF of *Boesenbergia rotunda* crude extract.

Independent Variables				Dependent Variables					
Point	SWE Condition			Y1, TPC			Y2, TF		
				(µg GAE/mg)			(µg GE/mg)		
Run	X_1_	X_2_	X_3_	Exp	Pred	DLM	Exp	Pred	DLM
	(M, % *w*/*v*)	(T (In), °C)	(T (Out), °C)						
1	20	140	80	14.97 ± 4.24 ^defg^	14.89	14.92	27.74 ± 3.95 ^e^	25.86	27.55
2	30	140	80	12.17 ± 0.81 ^bcde^	10.83	12.15	15.02 ± 2.14 ^c^	19.79	15.00
3	25	160	70	16.77 ± 6.22 ^efg^	12.45	16.77	11.63 ± 0.79 ^abc^	10.20	11.52
4	20	150	70	8.86 ± 0.40 ^abc^	15.34	8.81	13.63 ± 5.95 ^bc^	19.83	13.53
5	30	150	70	7.05 ± 1.07 ^ab^	11.27	6.97	7.27 ± 1.37 ^ab^	6.82	7.05
6	25	150	80	10.31 ± 1.85 ^abcd^	12.01	11.55	12.3 ± 3.79 ^abc^	10.01	9.87
7	25	150	80	13.78 ± 2.94 ^cdef^	12.01	11.55	8.94 ± 2.62 ^abc^	10.01	9.87
8	30	150	90	10.54 ± 0.46 ^abcd^	8.68	10.52	10.06 ± 1.97 ^abc^	3.86	10.03
9	20	160	80	18.96 ± 2.62 ^g^	13.19	18.60	33.52 ± 9.21 ^e^	28.75	33.23
10	25	140	90	11.97 ± 2.67 ^bcde^	11.57	11.96	6.63 ± 1.04 ^ab^	8.06	6.56
11	20	150	90	11.96 ± 0.68 ^bcde^	12.75	11.97	13.19 ± 3.32 ^bc^	13.64	13.03
12	30	160	80	8.72 ± 2.16 ^abc^	9.13	8.71	10.16 ± 1.44 ^abc^	12.04	10.02
13	25	160	90	5.63 ± 1.60 ^a^	9.87	5.55	5.55 ± 0.20 ^a^	9.87	5.57
14	25	150	80	10.7 ± 3.39 ^abcd^	12.01	11.55	8.8 ± 3.38 ^abc^	10.01	9.87
15	25	140	70	17.77 ± 2.47 ^fg^	14.15	17.75	21.21 ± 0.78 ^d^	16.89	20.96

Exp: Experimental values, performed in a random order and expressed as the average of triplicate determinations from different experiments (*n* = 3). Pred: Predicted values, based on BBD evaluation. DLM: Deep-Learning Machine. ^a–g^ Same letter show no significant difference at *p* < 0.05, different letter shows significant difference (*p* < 0.05) in the same column.

**Table 3:** Experimental and predicted values of DPPH, FRAP, and yield of *Boesenbergia rotunda* crude extract obtained by BBD.

Independent Variables				Dependent Variables								
Point	SWE Condition			Y3, DPPH			Y4, FRAP			Y5, Yield		
				(µg/mL)			(µmol Fe^2+^/mg)			(%)		
Run	X_1_	X_2_	X_3_	Exp ^a^	Pred ^b^	DLM ^c^	Exp ^a^	Pred ^b^	DLM ^c^	Exp ^a^	Pred ^b^	DLM ^c^
	(M, % *w*/*v*)	(T (In), °C)	(T (Out), °C)									
1	20	140	80	4.22 ± 0.12 ^d^	3.83	4.10	4.278 ± 0.77 ^fg^	4.30	4.281	36.4 ± 0.96 ^c^	35.22	36.42
2	30	140	80	2.95 ± 1.11 ^bcd^	1.98	2.93	3.216 ± 0.24 ^de^	3.60	3.220	62.72 ± 2.35 ^hi^	59.90	62.80
3	25	160	70	1.95 ± 0.41 ^abc^	2.73	1.93	3.219 ± 0.30 ^de^	3.21	3.221	52.41 ± 1.90 ^e^	51.87	52.53
4	20	150	70	3.21 ± 0.40 ^cd^	3.96	3.20	3.608 ± 1.04 ^ef^	4.00	3.610	45.17 ± 1.69 ^d^	35.67	45.20
5	30	150	70	0.67 ± 0.88 ^a^	2.11	0.70	2.911 ± 0.15 ^cde^	2.94	2.911	67.29 ± 0.46 ^j^	60.34	66.64
6	25	150	80	2.02 ± 0.64 ^abc^	2.60	3.02	1.28 ± 0.26 ^a^	1.84	1.840	57.23 ± 2.09 ^fg^	51.42	56.86
7	25	150	80	3.06 ± 0.38 ^cd^	2.60	3.02	2.161 ± 0.14 ^bc^	1.84	1.840	54.94 ± 1.65 ^ef^	51.42	56.86
8	30	150	90	1.44 ± 0.40 ^ab^	1.23	1.45	1.893 ± 0.22 ^ab^	1.50	1.890	60.93 ± 2.72 ^gh^	67.17	60.93
9	20	160	80	4.14 ± 0.92 ^d^	3.22	4.05	4.829 ± 0.75 ^g^	4.44	4.776	32.21 ± 2.07 ^b^	42.95	32.25
10	25	140	90	0.67 ± 0.25 ^a^	2.46	0.68	2.637 ± 0.52 ^bcd^	2.65	2.639	53.7 ± 1.15 ^ef^	50.98	53.70
11	20	150	90	3.03 ± 1.22 ^cd^	3.09	3.00	3.247 ± 0.27 ^de^	3.22	3.251	44.36 ± 2.11 ^d^	42.50	44.41
12	30	160	80	2.13 ± 1.52 ^abc^	1.37	2.11	2.38 ± 0.14 ^bcd^	2.36	2.380	65.89 ± 1.63 ^ij^	67.62	66.00
13	25	160	90	1.31 ± 0.68 ^a^	1.85	1.32	2.177 ± 0.30 ^bc^	2.59	2.181	56.55 ± 2.21 ^f^	58.70	56.60
14	25	150	80	4.05 ± 0.56 ^d^	2.60	3.02	2.076 ± 0.53 ^abc^	1.84	1.840	58.19 ± 0.68 ^fg^	51.42	56.86
15	25	140	70	4.13 ± 1.17 ^d^	3.34	4.02	4.666 ± 0.42 ^g^	4.25	4.672	23.34 ± 0.68 ^a^	44.14	23.46

^a^ Experimental values, performed in a random order and expressed as the average of triplicate determinations from different experiments (*n* = 3). ^b^ Predicted values, based on BBD evaluation. ^c^ Deep-Learning Machine. ^a–j^ Same letter show no significant difference at *p* < 0.05, different letter shows significant difference (*p* < 0.05) in the same column.

**Table 4:** Model summary and analysis of variance (ANOVA) of TPC and TF *Boesenbergia rotunda* crude extract.

Source	Sum of Squares	df	Mean Square	F-Value	*p*-Value		Source	Sum of Squares	df	Mean Square	F-Value	*p*-Value	
Model	52.26	3	17.42	1.22	0.3473	not significant	Model	705.63	9	78.40	2.19	0.2012	not significant
A-X_1_	33.09	1	33.09	2.32	0.1556		A-X_1_	259.58	1	259.58	7.24	0.0432	
B-X_2_	5.78	1	5.78	0.4059	0.5371		B-X_2_	11.86	1	11.86	0.3309	0.5900	
C-X_3_	13.39	1	13.39	0.9404	0.3530		C-X_3_	41.91	1	41.91	1.17	0.3289	
							AB	28.30	1	28.30	0.7898	0.4149	
							AC	2.61	1	2.61	4.87	0.7981	
							BC	18.06	1	18.06	2.74	0.5095	
							A^2^	119.53	1	119.53	1.88	0.1274	
							B^2^	128.84	1	128.84	1.64	0.1164	
							C^2^	80.37	1	80.37	2.39	0.1945	
Residual	156.62	11	14.24				Residual	179.18	5	35.84			
Lack of Fit	149.40	9	16.60	4.59	0.1915	not significant	Lack of Fit	171.32	3	57.11	14.54	0.0650	not significant
Pure Error	7.23	2	3.61				Pure Error	7.85	2	3.93			
Cor Total	208.88	14					Cor Total	884.81	14				
		Std.Dev = 3.77 R-Squared = 0.2502 Mean = 12.01 R-Squared = 0.0457 C.V. % = 3.42 Adeq Precision = 3.4153							Std.Dev = 5.99 R-Squared = 0.7975 Mean = 13.71 R-Squared = 0.4330 C.V. % = 43.66 Adeq Precision = 5.0921				

**Table 5:** Model summary and analysis of variance (ANOVA) of DPPH and FRAP *Boesenbergia rotunda* crude extract.

Source	Sum of Squares	df	Mean Square	F-Value	*p*-Value		Source	Sum of Squares	df	Mean Square	F-Value	*p*-Value	
Model	9.15	3	3.05	2.66	0.1002	not significant	Model	13.78	9	1.53	5.37	0.0394	significant
A-X_1_	6.86	1	6.86	5.98	0.0325		A-X_1_	3.87	1	3.87	13.56	0.0143	
B-X_2_	0.7442	1	0.7442	0.6486	0.4377		B-X_2_	0.6006	1	0.6006	2.11	0.2064	
C-X_3_	1.54	1	1.54	1.34	0.2712		C-X_3_	2.48	1	2.48	8.68	0.0320	
							AB	0.4809	1	0.4809	1.69	0.2507	
							AC	0.1079	1	0.1079	0.3785	0.5653	
							BC	0.2435	1	0.2435	0.8542	0.3978	
							A^2^	2.29	1	2.29	8.05	0.0364	
							B^2^	4.06	1	4.06	14.23	0.0130	
							C^2^	0.3049	1	0.3049	1.07	0.3485	
Residual	12.62	11	1.15				Residual	1.43	5	0.2851			
Lack of Fit	10.56	9	1.17	1.14	0.5516	not significant	Lack of Fit	0.9532	3	0.3177	1.35	0.4532	not significant
Pure Error	2.06	2	1.03				Pure Error	0.4723	2	0.2362			
Cor Total	21.77	14					Cor Total	15.21	14				
		Std.Dev = 1.07 R-Squared = 0.4202 Mean = 2.60 R-Squared = 0.2621 C.V. % = 41.22 Adeq Precision = 4.9353							Std.Dev = 0.5339 R-Squared = 0.9063 Mean = 2.97 R-Squared = 0.7376 C.V. % = 17.97 Adeq Precision = 6.7545				

**Table 6:** Model summary and analysis of variance (ANOVA) of %yield *Boesenbergia rotunda* crude extract.

Source	Sum of Squares	df	Mean Square	F-Value	*p*-Value	
Model	1430.18	3	476.73	6.20	0.0101	significant
A-X_1_	1217.46	1	1217.46	15.84	0.0022	
B-X_2_	119.35	1	119.35	1.55	0.2387	
C-X_3_	93.37	1	93.37	1.21	0.2940	
Residual	845.68	11	76.88			
Lack of Fit	840.10	9	93.34	33.48	0.0293	significant
Pure Error	5.58	2	2.79			
Cor Total	2275.86	14				
		Std.Dev = 8.77 R-Squared = 0.6384 Mean = 51.42 R-Squared = 0.5271 C.V. % = 17.05 Adeq Precision = 7.1552				

## Data Availability

The original contributions presented in the study are included in the article, further inquiries can be directed to the corresponding authors.
